# A Comparison of 11 Clinical Risk Scores for Prediction of Survival After Curative-Intent Resection of Colorectal Liver Metastases

**DOI:** 10.1245/s10434-026-19627-9

**Published:** 2026-04-27

**Authors:** Felix Schmidt, Franziska A. Meister, Lea Hitpass, Theresa H. Wirtz, Sven A. Lang, Felix Oldhafer, Oliver Beetz, Martin W. von Websky, Thomas Vogel, Florian W. R. Vondran, Katharina Joechle, Iakovos Amygdalos

**Affiliations:** 1 Department of General, Visceral, Pediatric, and Transplantation Surgery, University Hospital RWTH Aachen, Aachen, Germany; 2 Department of Diagnostic and Interventional Radiology, University Hospital RWTH Aachen, Aachen, Germany; 3 Department of Internal Medicine III, University Hospital RWTH Aachen, Aachen, Germany; 4 Department of General, Visceral, Thoracic, and Endocrine Surgery, Johannes Wesling University Hospital Minden, Ruhr University Bochum, Minden, Germany

**Keywords:** Colorectal liver metastases, Clinical risk scores, Survival, Prognosis

## Abstract

**Background:**

Colorectal liver metastases (CRLM) are the main determinant of survival in colorectal cancer, and radical resection offers the best oncological outcomes. However, heterogeneous clinicopathological features make appropriate patient selection essential. Numerous clinical risk scores (CRS) have been proposed to predict outcomes after liver resection for CRLM. This study evaluated the prognostic performance of 11 established CRS regarding survival after curative-intent resection of CRLM.

**Methods:**

This retrospective study included patients who underwent curative-intent liver resection for CRLM at University Hospital RWTH Aachen, Germany, between 2010 and 2021. The following CRS were analyzed: Fong, Nordlinger, Nagashima, Konopke, Basingstoke Predictive Index, Tumor Burden Score, Resection Severity Index, Kulik, RAS-mutation CRS, Comprehensive Evaluation of Relapse Risk (CERR) score, and the Genetic and Morphological Evaluation score. Overall survival (OS) was compared using Kaplan–Meier analysis and log-rank testing. Predictive accuracy was assessed using the Akaike information criterion, Harrell’s C-index for OS, and area under the curve (AUC) analyses for 1- and 5-year survival.

**Results:**

A total of 528 patients were included, with a median OS of 26 months (95% confidence interval [CI] 23–28). All CRS except the Resection Severity Index significantly stratified patients according to OS. CERR consistently ranked among the top three scores for both the Akaike information criterion (1725) and the C-index (0.61) and had the highest accuracy for predicting 1-year survival (AUC 0.654, *p *= 0.001) and 5-year survival (AUC 0.62, *p *< 0.001).

**Conclusions:**

Although the CERR demonstrated the most consistent predictive performance, 10 of 11 evaluated CRS effectively stratified patients according to long-term survival after CRLM resection.

**Supplementary Information:**

The online version contains supplementary material available at 10.1245/s10434-026-19627-9.

Up to 75% of patients with colorectal cancer develop liver metastases, which are the most predominant factor limiting survival.^1,2^ Although surgical resection offers the best curative option for these patients, 5-year overall-survival (OS) rates of up to 60% remain considerably low, whereas metastatic recurrence rates range between 40 and 75% after liver resection.^3^ As patients present with varying clinicopathological and oncological characteristics, careful planning of treatment strategies and preoperative patient selection are essential for optimal oncological outcomes.^4^ To this end, several clinical risk scores (CRS) have been developed to predict OS and recurrence-free survival.

Most established CRS use cut-off values to divide patients into different risk groups. For example, the CRS by Nordlinger et al.^5^, Fong et al.^6^, Schindl et al.^7^, Konopke et al.^8^, and others use a “categorical approach”, differing mostly in the selection of variables and cut-off values. A different approach was first introduced with the Basingstoke Predictive Index (BPI), where variables were weighted based on their influence on OS.^9^ Additionally, Sasaki et al.^10^ conceived the tumor burden score (TBS), based on a novel “metro ticket” paradigm, which decreases the risk of bias. Finally, recently developed CRS include genetic information, such as described by Brudvik et al.^11^, which focuses on rat sarcoma viral oncogene homolog (RAS) mutation status. Moreover, the Genetic and Morphological Evaluation (GAME), and Comprehensive Evaluation of Relapse Risk (CERR) CRS combined TBS with genetic information.^12,13^

To date, few studies have compared different risk scores in patients undergoing liver resection for CRLM. For example, Merkel et al.^14^ compared the Fong and Nordlinger scores, demonstrating that only the Fong score could predict OS. Additionally, Reissfelder et al.^15^ analyzed five CRS (Nordlinger, Fong, Iwatsuki, BPI, and Mayo scoring index scores) and found the Fong and Iwatsuki CRS capable of significant stratification in predicting survival. Finally, Bolhuis et al.^16^ compared the Fong and GAME CRS, with both reaching significance in predicting OS. In contrast, previous work from our group focused on recurrent CRLM. Although various CRS could also be successfully applied to this cohort of patients, the Nagashima CRS showed the best prognostic stratification.^3^

The aforementioned studies only compared a small number of CRS or included relatively small patient cohorts. Thus, the aim of this study was to identify the best predictive CRS for OS after curative-intent resection of CRLM on a larger sample. To this end, we conducted a comprehensive comparison of 11 CRS, including both long-established and more recently developed scores.

## Methods

### Study Population

All patients who underwent elective, curative-intent liver resection for CRLM at the University Hospital RWTH Aachen between 2010 and 2021 were retrospectively evaluated from a prospectively maintained database. Exclusion criteria included missing data, resection of recurrent metastases, and death within 90 days after surgery. The latter was considered part of perioperative complications and therefore not relevant for assessment of the included CRS, which are designed for prediction of longer-term survival. The retrospective study design meant that race/ethnicity data of the study population were not available.

### Oncological Indication and Operative Technique

After completion of appropriate preoperative staging examinations, such as computed tomography of the chest and abdomen and liver-specific magnetic resonance imaging, patients were discussed in the institutional multidisciplinary tumor board.

Intraoperatively, imaging findings were confirmed, and possible new manifestations excluded with sonography. Parenchymal transection was performed with the Cavitron Ultrasonic Surgical Aspirator (CUSA®, Integra LifeSciences, Plainsboro, NJ, USA) in open surgery, and vessel-sealing devices were combined with vascular staplers in minimally invasive approaches. Intermittent inflow occlusion (Pringle’s maneuver) was used as required. During parenchymal transection, low central venous pressure was maintained through restrictive fluid management strategies. Tumor margins were controlled by intraoperative frozen section. Postoperatively, further treatment and/or follow-up was discussed in the multidisciplinary tumor board according to national and international guidelines. For patients who underwent concomitant local ablation therapy, additional liver magnetic resonance imaging was performed every 3 months for 2 years and afterwards increased to a 6-month time interval.

### Data Collection

Our prospectively maintained institutional database includes demographic and clinicopathological data, histological information (including mutation status, where available), TNM classification, laboratory results (including tumor markers such as carcinoembryonic antigen [CEA]), radiological and staging information (such as extrahepatic metastases), operative variables, postoperative complications (classified according to Clavien–Dindo and comprehensive complication index), as well as long-term survival and recurrence outcomes. Relevant variables were selected to stratify patients according to the 11 predictive CRS compared in this study. Where variables were missing, efforts were made to retrospectively recover the necessary information or the patients were excluded. No imputation or other statistical techniques for missing data were employed.

### Calculation of Predictive Scores

In total, 11 CRS were compared, namely Nordlinger,^5^ Fong,^6^ Nagashima,^17^ BPI,^9^ Konopke,^8^ Resection severity index (RSI),^18^ TBS,^10^ GAME,^12^ Kulik,^19^ RAS-mutation CRS,^11^ and CERR.^13^ The majority of these scoring systems are calculated in a straightforward manner: one point is assigned for each risk factor. Based on the number of points, patients are assigned to different risk groups, and higher scores indicate a higher risk of adverse outcomes. A lack of relevant data or outdated risk variables meant that not every published CRS could be considered in this project, such as those described by Iwatsuki et al.^20^ and Schindl et al.^7^. Details regarding score parameters and their interpretation are listed in Table 1 and Supplementary Table 1.

**Table 1:** Overview of all clinical risk scores (CRS)

CRS	Criteria	Predicted outcome	Risk groups
Nordlinger et al. ^5^, 1996	Age >60 years Size of largest metastasis >5 cm Serosal invasion of primary tumor (>pT3) Disease-free interval <24 months Lymph node-positive primary tumor Number of liver nodules >3	2-year survival	Low = 0–2 points Intermediate = 3–4 points High = 5–6 points
Fong et al. ^6^, 1999	Lymph node-positive primary tumor Disease-free interval to metastases <12 months Number of hepatic tumors >1 Largest hepatic tumor >5 cm CEA >200 ng/ml	5-year survival	Low: 0–2 points High: 3–5 points
Nagashima et al. ^17^**, **2004	Serosa invasion of primary tumor (>pT3) Lymph node-positive primary tumor Multiple nodules of hepatic metastases ≥2 Hepatic metastases >5 cm Resectable extrahepatic distant metastases	Cancer-free survival	Low: 0–1 point Intermediate: 2–3 points High: ≥4 points
Rees et al. ^9^, 2008/BPI	Number of hepatic metastases >3 Node-positive primary Poorly differentiated primary Extrahepatic disease Tumor diameter ≥5 cm CEA >60 ng/mL Positive hepatic resection margin (1–6 = preoperative score, 2–7 = postoperative score)	Cancer-specific survival (5-year survival)	0–5 points 6–10 points 11–15 points 6–20 points 21–25 points 26–30 points (for details, see Supplementary Table 1)
Konopke et al. ^8^, 2009	Synchronous manifestation Number of liver metastases ≥4 Increased preoperative CEA level ≥200 ng/mL	5-year survival/OS 5-year RFS	Low = 0 points Intermediate = 1 point High ≥2 points
Gwiasda et al. ^18^, 2017/RSI	AST/GOT Quick (in percentage) Extent of liver resection (graded in points) ${\mathrm{RSI}} = \frac{{{\mathrm{AST}}}}{{{\mathrm{Quick}}}}{\text{*extent of liver resection}}$	Cancer mortality in 5–10 years	RSI 1: 1–25% RSI 2: 26–50% RSI 3: 51–75% RSI 4: 76–100%
Sasaki et al. ^10^, 2018/TBS	${\mathrm{TBS}}^{2}={\left(\text{maximum tumor diameter}\right)}^{2}+{\left(\text{number of liver lesions}\right)}^{2}$	5-year survival	Zone 1 = lowest 25% → <3 points Zone 2 = 25^th^–90^th^ % → ≥3 to <9 points Zone 3 = highest 10% → ≥ 9 points
Margonis et al. ^12^, 2018/GAME	KRAS-mutated tumors (primary tumor) = 1 point CEA ≥20 ng/ml = 1 point Primary tumor lymph node metastasis = 1 point 3 ≤ TBS < 9 = 1 point, TBS ≥9 = 2 points Extrahepatic disease = 2 points	5-year survival	Low = 0–1 point Medium = 2–3 points High ≥4 points
Kulik et al. ^19^, 2018	Age at liver resection Chemotherapy for the primary tumor Preoperative Quick Hemoglobin level Grading of primary colorectal tumor (≥G3) 10-year mortality risk (%) = 1/(1 + Exp_(-y)_)	10-year survival	No specific risk groups defined
Brudvik et al. ^11^, 2019/RAS-Mutation CRS	RAS mutation status Positive primary lymph node status (N1) Largest liver metastases >5 cm	5-year survival RFS	0 points 1 point 2 points 3 points
Chen et al. ^13^, 2020/CERR	KRAS / NRAS / BRAF-mutated tumor = 1 point Node-positive primary = 1 point Extrahepatic disease = 1 point CEA >200 ng/ml or CA19-9 >200 U/ml mTBS 5–11 = 1 point mTBS ≥12 = 2 points formula mTBS: mTBS_unilobar disease_ = $\sqrt {{\mathrm{e}}^{0} {\text{*maximum CRLM diameter}}^{2} + {\text{number of CRLM}}^{{2}} }$ mTBS_bilobar disease_ =$\sqrt {{\mathrm{e}}^{1} {\text{*maximum CRLM diameter}}^{2} + {\text{number of CRLM}}^{{2}} }$	RFS	Low: 0–1 point Medium: 2–3 points High ≥4 points

AST, aspartate transaminase; BPI, Basingstoke Predictive Index; CA19-9, carbohydrate antigen 19-9; CEA, carcinoembryonic antigen; CERR, Comprehensive Evaluation of Relapse Risk; CRLM, colorectal liver metastasis; GOT, glutamic oxaloacetic transaminase; mTBS, ; OS, overall survival; RFS, recurrence-free survival; RSI, Resection Severity Index; TBS, tumor burden score

### Statistical Analysis

SPSS, version 27 (IBM Corp., Armonk, NY, USA) and Microsoft Excel (Microsoft Corp., Redmond, WA, USA) were used for statistical analysis. Categorical variables were expressed as absolute values and percentages, whereas continuous variables were presented as medians and interquartile ranges (IQRs). OS was calculated from the date of last surgery to the date of last follow-up or death. The Kaplan–Meier method was used to graphically depict survival rates for each score, and OS was compared between groups by log rank test. Reverse Kaplan–Meier was used to calculate the median follow-up time. Comparisons of predictive ability between the CRS included in this study were based on OS since it is often considered the “gold-standard” in oncology.^21^

Since Kulik’s CRS predicts 10-year survival for each patient individually, without stratification into risk groups, it could not be assessed using the Kaplan–Meier method. The Akaike information criterion (AIC),^22^ Harrell’s C-Index,^23^ and area under the curve (AUC) analysis were used to determine the best CRS regarding data fit and predictive performance.

The AIC is used to compare model fits and favors more parsimonious models. It is calculated using the likelihood ratio and number of parameters of the (regression) model. Lower AIC values indicate a better model fit. Harrell’s C-Index evaluates the proportion of concordant pairs out of all possible patient pairs. A higher proportion is represented by a higher C-index value, which indicates a better model fit. AIC and Harrell’s C-Index were calculated for OS, and the AUC analysis was conducted on 1-year and 5-year survival. P values <0.05 were considered statistically significant.

## Results

### Patient Characteristics

Of 550 eligible patients who underwent surgery during the study period, 528 were included in this study. The median age was 62 years, and 60% of patients were male. Metastases were mainly synchronous (67%) and bilateral (57%), and most patients received perioperative chemotherapy (65%). The median diameter of the largest lesion was 3 cm (IQR 1.8–4.7). Major liver resection was performed in 269 patients (49%). An R0 resection margin was achieved in 465 patients (88%). Patient and tumor characteristics are listed in Table 2.

**Table 2:** Demographic and perioperative characteristics of the study cohort

Patient demographics	*n* (total = 528)
Age, years	62 (55–70)
Male sex	314 (60)
ASA I	14 (3)
ASA II	204 (39)
ASA III	290 (55)
ASA IV	20 (4)
BMI, kg/m^2^	25 (23–29)
Primary tumor location colon	297 (56)
Primary tumor location rectum	231 (44)
Serosal invasion of primary tumor (>pT3)	101 (19)
Nodal positive primary^a^	333 (63)
Synchronous disease	352 (67)
Major liver resection	269 (51)
Type of resection according to Brisbane classification Atypical resections Sectorectomy (left and right) Left hemihepatectomy Right hemihepatectomy Extended left hepatectomy Extended right hepatectomy	212 (40) 93 (18) 39 (7) 105 (20) 10 (2) 59 (11)
ALPPS procedures	18 (3)
Preoperative PVE	106 (20)
Extent of liver resection (RSI) ^b^ 1 (atypical resection) 2 (segmental resection) 3 (left hemihepatectomy) 4 (right hemihepatectomy) 5 (extended left hepatectomy) 6 (extended right hepatectomy)	212 (40) 93 (18) 39 (7) 105 (20) 10 (2) 59 (11)
Number of liver metastases	3 (1–5)
Bilateral liver metastases	303 (57)
Largest diameter of liver metastases, cm	3 (1.8–4.7)
Number of liver metastases >3	206 (39)
KRAS mutation^c^	128 (24)
NRAS mutation^c^	23 (4)
BRAF mutation^d^	14 (3)
Preoperative CEA, ng/ml^e^	8.6 (3.6–34.7)
Preoperative hemoglobin, g/dl	12.9 (11.8–14.2)
Preoperative INR	0.98 (0.94–1.03)
Preoperative AST/GOT	29.0 (23.0–39.0)
Chemotherapy (neoadjuvant or adjuvant)^f^	345 (65)
Extrahepatic disease	101 (19)
Operative time, minutes^g^	227 (167–298)
Intraoperative transfusion red cell concentrates	133 (25)
Fresh frozen plasma	162 (31)
R0 resection for liver metastases^h^	465 (88)
Postoperative complications Clavien–Dindo ≥3b	86 (16)
CCI	20.9 (8.7–34.6)
Disease-free survival <12 months	250 (47)
Disease-free survival <24 months	69 (13.1)

Data are presented as n (%) or median (interquartile range) unless otherwise indicated

^a^Missing for 46 patients, ^b^Missing for seven patients, ^c^Missing for 191 patients, ^$^Missing for 308 patients, ^d^Missing for 384 patients, ^e^Missing for 130 patients, ^f^Missing for six patients, ^g^Missing for 173 patients, ^h^Missing for two patients

ALPPS, associating liver partition with portal vein ligation for staged hepatectomy; AST, aspartate transaminase; CCI, Comprehensive Complication Index; CEA, carcinoembryonic antigen; BMI, body mass index; GOT, glutamic oxaloacetic transaminase; INR, international normalized ratio; PVE, portal vein embolization; RAS, rat sarcoma viral oncogene homolog; KRAS, Kirsten rat sarcoma virus; NRAS, neuroblastoma RAS viral oncogene homolog; RSI, Resection Severity Index

### Survival Analysis

**Fig. 1:**
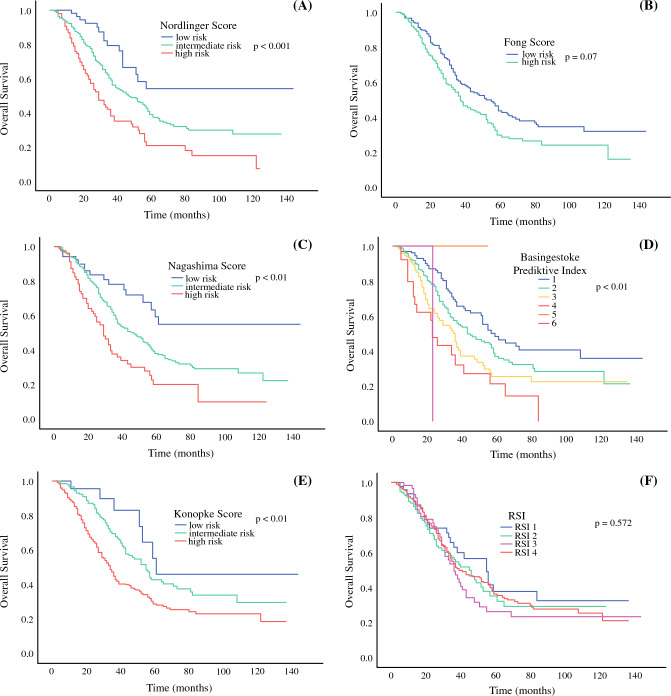

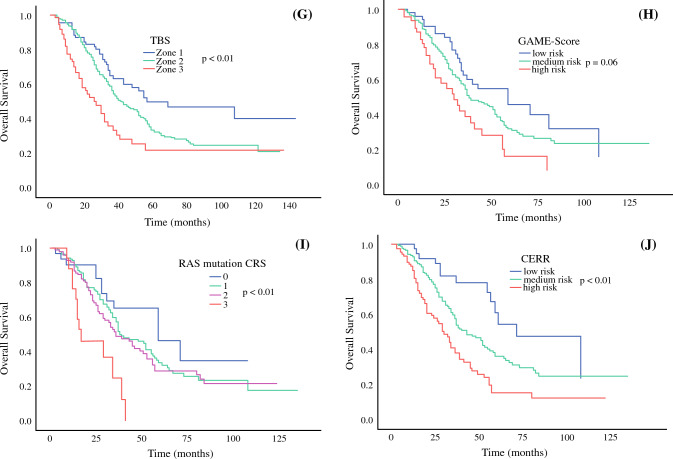
Kaplan–Meier curves for overall survival of patient risk groups, as stratified by different clinical risk scores (CRS). CERR, Comprehensive Evaluation of Relapse Risk; GAME, Genetic and Morphological Evaluation; RSI, Resection Severity Index; TBS, tumor burden score

Median follow-up time was 51 months, with a 1-year survival rate of 93% and a 5-year survival rate of 56%. Median OS was 41 months (range 0–144). All CRS, except for the RSI, were able to stratify patients into risk groups with statistically significant differences in OS (Fong: *p *= 0.007; Nordlinger: *p *< 0.001; GAME: *p *= 0.006; Nagashima: *p *< 0.001; Konopke: *p *< 0.001; BPI: *p *< 0.001; TBS: *p *< 0.001; RSI: *p *= 0.572; Kulik: *p *< 0.001; RAS-mutation-CRS: *p *< 0.001; CERR: *p *< 0.001). Survival differences between CRS-stratified risk groups are depicted as Kaplan–Meier curves in Fig. 1. Data regarding patient stratification and survival outcomes according to each CRS are outlined in Table 3.

**Table 3:** Stratification of patients into risk groups and corresponding survival outcomes according to each clinical risk score (CRS)

Score			Total *n *= 528 (100%)		Median OS (95% CI)	*p*-Value
Nordlinger risk score	0	2 (0.4%)	Low risk 0–2	72 (13.6%)	N/A	<0.001
	1	16 (3.0%)				
	2	54 (10.2%)				
	3	141 (26.7%)	Intermediate risk 3–4	301 (57.0%)	47 (37–57)	
	4	160 (30.3%)				
	5	106 (20.1%)	High risk 5–6	118 (22.3%)	29 (22–36)	
	6	12 (2.3%)				
Fong's CRS	0	13 (2.5%)	Low risk 0–2	241 (57.7%)	55 (45–65)	0.007
	1	83 (15.7%)				
	2	145 (27.5%)				
	3	186 (35.2%)	High risk 3–5	234 (45.2%)	37 (31–44)	
	4	44 (8.3%)				
	5	4 (0.8%)				
Nagashima	0	7 (1.3%)	Low risk 0–1	59 (11.2%)	N/A	<0.001
	1	52 (9.8%)				
	2	136 (25.8%)	Intermediate risk 2–3	337 (63.8%)	46 (37–55)	
	3	201 (38.1%)				
	4	76 (14.4%)	High risk ≥4	89 (16.9%)	29 (23–35)	
	5	13 (2.5%)				
Basingstoke Predictive Index	1	145 (27.5%)			59 (45–73)	<0.001
	2	201 (38.1%)			43 (31–55)	
	3	104 (19.7%)			36 (29–43)	
	4	29 (5.5 %)			23 (17–29)	
	5	2 (0.4%)			(N/A)	
	6	1 (0.2%)			23	
Konopke's CRS	0	29 (5.5%)	Low risk 0	29 (5.5%)	61 (N/A)	<0.001
	1	171 (32.4%)	Intermediate risk 1	171 (32.4%)	55 (47–63)	
	2	209 (39.6%)	High risk ≥2	328 (62.1%)	34 (30–38)	
	3	119 (22.5%)				
RSI	1	49 (9.3%)			55 (40–70)	0.572
	2	139 (26.3%)			46 (34–58)	
	3	68 (12.9%)			36 (30–43)	
	4	267 (50.6%)			39 (28–50)	
Tumor burden score	Zone 1	101 (19.1%)			57 (13–101)	<0.001
	Zone 2	347 (65.7%)			42 (34–50)	
	Zone 3	80 (15.2%)			27 (18–36)	
GAME score	0	7 (1.3%)	Low risk 0–1	56 (10.6%)	59 (29–89)	0.006
	1	49 (9.3%)				
	2	118 (22.3%)	Intermediate risk 2–3	204 (38.6%)	38 (29–47)	
	3	86 (16.3%)				
	4	37 (7.0%)	High risk ≥4	49 (9.3%)	30 (20–40)	
	5	11 (2.1%)				
	6	1 (0.2%)				
	7	0 (0%)				
	8	0 (0%)				
RAS mutation CRS	0	35 (6.6%)			59 (29–90)	<0.001
	1	150 (28.4%)			39 (28–50)	
	2	105 (19.9%)			36 (29–41)	
	3	17 (3.2%)			17 (5–29)	
CERR score	0	6 (1.1%)	Low risk 0–1	40 (7.6%)	71 (41–101)	<0.001
	1	34 (6.4%)				
	2	82 (15.5%)	Intermediate risk 2–3	186 (35.2%)	43 (35–51)	
	3	104 (19.7%)				
	4	71 (13.4%)	High risk 4–7	92 (17.4%)	30 (24–36)	
	5	15 (2.8%)				
	6	6 (1.1%)				

Kulik is not depicted because no risk groups were defined

Missing values: Fong’s CRS 53 (10.0%), Nordlinger’s risk score 37 (7.0%), Nagashima 43 (8.1%), RAS-mutation CRS 221 (41.9%), Konopke’s CRS 0, BPI 49 (8.9%), TBS 0, GAME 219 (41.5%), RSI 5 (0.9%), CERR 210 (39.8%).

BPI, Basingstoke Predictive Index; CERR, Comprehensive Evaluation of Relapse Risk; CI, confidence interval; GAME, Genetic and Morphological Evaluation; N/A, not applicable; OS, overall survival; RSI, Resection Severity Index; TBS, tumor burden score

### Comparison of the Different CRS

According to AIC analysis, the RAS-mutation, GAME, and CERR scores performed best (AIC values of 1670, 1682, and 1725, respectively). Furthermore, Harrel’s C-Index stratified the CERR, BPI, and Nordlinger CRS as the most accurate in predicting OS (C-Indices of 0.61, 0.60, and 0.60, respectively). Regarding 1-year-survival, the TBS (AUC 0.65, *p *= 0.001), CERR (AUC 0.65, *p *< 0.001), and Nordlinger CRS (AUC 0.63, *p *= 0.009) demonstrated the best predictive capabilities. On the other hand, 5-year-survival rates were most accurately forecasted by the CERR (AUC 0.62, *p *< 0.001), the Nordlinger CRS (AUC 0.60, *p *< 0.001), and the BPI (AUC 0.60, *p *< 0.001). Throughout the different analyses, RSI and Kulik’s CRS consistently had the worst predictive performance. Detailed results are outlined in Table 4.

**Table 4:** Prediction accuracy metrics for each clinical risk score (CRS)

Score	AIC	C-Index	AUC 12 Months	*p*-Value	AUC 60 months	*p*-Value
Nordlinger	2429	0.60	0.63	0.009	0.60	<0.001
Fong	2399	0.55	0.57	0.191	0.56	0.025
Nagashima	2401	0.57	0.59	0.072	0.58	0.002
Basingstoke Predictive Index	2388	0.60	0.62	0.025	0.60	<0.001
Konopke	2690	0.59	0.62	0.012	0.57	0.005
Resection Severity Index	2698	0.50	0.43	0.275	0.54	0.225
TBS	2692	0.58	0.65	0.001	0.56	0.021
GAME score	1682	0.57	0.59	0.154	0.56	0.057
Kulik	2065	0.46	0.45	0.347	0.46	0.172
RAS mutation CRS	1670	0.57	0.53	0.685	0.58	0.019
CERR	1725	0.61	0.68	0.021	0.62	<0.001

AIC, Akaike information criteria; AUC, area under the curve; CERR, Comprehensive Evaluation of Relapse Risk; GAME, Genetic and Morphological Evaluation; TBS, tumor burden score

## Discussion

In this study, we compared CRS for prediction of survival in patients undergoing curative-intent resection of CRLM. Our aim was to identify the most accurate model in the era of multimodal treatment strategies. Using a large retrospective single-center cohort, we assessed the discriminatory ability of each CRS and showed CERR to be the most accurate across different survival outcomes. To our knowledge, this is the first study to comprehensively compare 11 CRS in a large cohort of patients with CRLM.

All but one CRS significantly stratified patients into prognostic groups. Across multiple analyses—including AIC, C-index, and AUC—the CERR score had the best performance, and the RSI was the least accurate and the only score unable to distinguish survival outcomes. The superiority of the CERR likely reflects its inclusion of modern prognostic variables, particularly the mutation status of KRAS, NRAS, and BRAF.

Although important prognostic markers such as “CEA level” or “node-positive primary” have already been included in early scoring systems (e.g. Fong et al.^6^) and used by more modern CRS, incorporating BRAF mutations appears to enhance prediction, consistent with current treatment approaches increasingly guided by molecular profiles.^24,25^

In contrast, the poor performance of the RSI in this study could be because of differences in sample characteristics analyzed between the original and present studies. For example, the proportion of patients receiving adjuvant chemotherapy (65% vs. 31%) may reduce performance by masking the prognostic value of variables such as lymph node status and serosa infiltration.^26^ Moreover, the score distribution differed between the two samples (mean RSI 1.6 in the original study vs. 3.06 in our sample). This suggests that our sample had worse prognostic factors, likely leading to a poorer outcome. Another contributing factor may be the compromised accuracy in calculating the variable "extent of liver resection". First, the divergence in study periods between Gwiasda et al.^18^ (2000–2014) and the present analysis (2010–2021) may have introduced a definitional bias. Surgical innovations and evolving treatment strategies – most notably the introduction of the ALPPS (Associating liver partition with portal vein ligation for staged hepatectomy) procedure in 2007^27^ – were only partially adopted during the earlier study period, potentially leading to inconsistencies in how the extent of liver resection was classified and recorded. Second, the encoding of this variable leaves room for interpretation. For instance, patients receiving a right hepatectomy would score 4 points based on the RSI calculation. However, patients often receive a hemihepatectomy and a contralateral atypical liver resection simultaneously (7.4% of patients in our study). For these patients, how “the extent of liver resection” should be graded remains unclear. Both can lead to variations in the score calculation.

Previous studies have investigated the predictive capability of established CRS on patients with CRLM, with varying methodologies and results. For example, Merkel et al.^14^ compared the Fong and Nordlinger CRS in 282 patients with 303 liver resections. In addition, the TNM classification was evaluated on its predictive ability on survival. Fong’s and Nordlinger’s CRS reached significance, whereas the TNM classification did not.^14^ Reissfelder et al.^15^ conducted a similar study and compared the accuracy of the CRS by Nordlinger, Fong, and Iwatsuki, the BPI, and the Mayo scoring index on a sample of 281 patients. In their study, only the CRS by Fong and Iwatsuki discriminated patients significantly.^15^ Both studies were conducted during a time span in which more complex surgical approaches were not yet accessible (Merkel et al.^14^ 1995–2006, Reissfelder et al.^15^ 2002–2008). Moreover, only small cohorts were evaluated. But mostly, both studies focused on validation of the CRS, without comparing their predictive abilities. Bolhuis et al.^16^ tried to answer this question by comparing the discriminatory ability of the Fong and GAME CRS in 1105 patients from 29 different hospitals. Like us, these authors employed Harrell’s C-index to assess model performance, and their reported values fell within a range comparable to ours (Fong: 0.58 vs. 0.55; GAME: 0.60 vs. 0.57).^16^ However, our study incorporated AIC and AUC analysis, strengthening the robustness and validity of our comparative evaluation. Finally, Ayez et al.^28^ compared the Nordlinger, Fong, Nagashima, and Konopke CRS in patients who underwent liver surgery directly (n=193) and those who underwent preoperative chemotherapy (n=159). In the second group, only the Nordlinger score was effective before chemotherapy, whereas all but the Konopke score showed good predictive ability after treatment.^28^ The main variables changed by neoadjuvant therapy were the number and size of metastases and the serum CEA level. In our study, we calculated CRS based on the latest images and laboratory results before surgery so the effects of perioperative chemotherapy were also accounted for. Compared with Ayez et al.^28^, we compared more CRS, including newer iterations accounting for tumor biology, and our study cohort included patients undergoing multimodal therapies and aggressive surgical techniques, such as ALPPS.

Although CRS are useful tools for prediction of patient outcomes, they lack enough discriminatory ability to reliably support clinical decision-making.^16^ As surgery is generally the treatment of choice for technically and oncologically resectable CRLM in patients fit enough to undergo liver resection, CRS may not always be used in treatment planning. Nonetheless, there are several reasons to consider the potential clinical utility of CRS. First, they may facilitate patient communication, enabling individuals to better understand their personalized prognosis and to incorporate this information into shared decision-making regarding treatment options. Furthermore, CRS could also assist in identifying high-risk patients who may benefit from intensified management strategies, such as additional preoperative diagnostics, neoadjuvant chemotherapy, or closer postoperative surveillance. For example, Grobmyer et al.^29^ applied Fong’s CRS to select patients for diagnostic laparoscopy. Similarly, Ivanecz et al.^30^ used Fong’s CRS to identify patients suitable for either immediate hepatic resection or additional chemotherapy. Konopke et al.^8^ used their score preoperatively to identify patients who could benefit from neoadjuvant chemotherapy. Building on the results of our study, similar prospective studies using the CERR score could be carried out to evaluate its usefulness in treatment guidance.

Certain limitations of this study need to be considered. First, its retrospective and monocentric design leads to selection bias and missing data (including outcomes), which are often impossible to recover. Second, the CRS compared were developed during different time periods. Modern treatment approaches, which were not available when these older CRS were developed, might explain why some older scores were less accurate in predicting survival of a study cohort. Third, not all CRS were designed to predict the same outcome (e.g. 5-year survival, OS, cancer-free survival), which limits direct comparability.

Future prospects for outcome prediction in CRLM mostly center around machine learning (ML) approaches. The first group to introduce a CRS for CRLM based on ML was Spelt et al.^31^. Similar approaches using different ML models have been taken by Paredes et al.^32^, Lam et al.^33^, and our workgroup.^34^ Despite differences in the algorithms and variables included in the CRS, all of these showed good discriminatory ability for various oncological outcomes. However, although ML models can analyze large amounts of data, they have the same drawbacks as the “traditional” CRS, namely retrospective study design, small sample sizes, and mainly internal validation.^35^ In addition, there is a risk of “overfitting”, meaning that the model is well trained on the existing data and thus cannot make reliable predictions on new data. Moreover, some of these models offer online calculators, where information can be entered to receive risk assessments for individual patients, and others do not. The type of prediction made also varies between models, and the lack of standardized point scores makes them hard to compare with traditional CRS. For these reasons, we refrained from including them in this study. All in all, ML-based methodologies offer the potential to generate more nuanced CRS. Nonetheless, their clinical utility remains to be confirmed through rigorous validation and prospective studies.

A further area of increasing importance in outcome prediction is tumor biology, which has a major impact on oncological outcomes in patients with CRLM. For example, RAS/RAF mutations are associated with worse OS and recurrence-free survival and an increased rate of extrahepatic metastases and recurrences in patients undergoing liver resection for CRLM.^36^ High microsatellite instability leads to resistance to 5-fluorouracil–based chemotherapies, and the interplay between BRAF mutations, high microsatellite instability, and poor prognosis is of great interest.^36^ Other relevant gene mutations include TP53, APC, PIK3CA, and SMAD4. Although the effects of individual mutations remain unclear, combinations of these mutations are associated with worse oncological outcomes.^36–38^ As mentioned, newer CRS (such as CERR) include tumor biology, and this is likely to be the standard for all prediction algorithms in future.

As newer prediction models emerge, encompassing artificial intelligence or genetic information or both, and multimodal treatment strategies become increasingly sophisticated,^39–41^ the question arises: are older CRS becoming irrelevant? To answer this, similar comparative studies must be conducted in large multicentric cohorts encompassing newer models as they arise. At the same time, well-designed prospective studies to develop effective ML models are necessary, including genetic information, radiological images, digital histology, and physiological parameters.^2,35,42^ The future will show whether newer CRS render the old ones obsolete.

## Conclusions

To our knowledge, this is the first study to comprehensively compare 11 CRS for the prediction of survival after curative-intent resection of CRLM. All but one accurately stratified patients according to postoperative survival. Across multiple statistical methods and outcomes, the CERR CRS emerged as the most consistent performer. These findings can be used to empower patients with decision-making for their own health and help doctors with treatment planning.

## Supplementary Information

Below is the link to the electronic supplementary material.

Supplementary file1 (DOCX 18 KB)
