# Supplementary material for: A Comparison of 11 Clinical Risk Scores for Prediction of Survival After Curative-Intent Resection of Colorectal Liver Metastases
Source: Ann Surg Oncol. 2026 Apr 27;33(8):7046–56. doi: 10.1245/s10434-026-19627-9 (PMC13337928; doi:10.1245/s10434-026-19627-9)
Supplement: Supplementary file 1 — Supplementary file1 (DOCX 18 KB) [file 10434_2026_19627_MOESM1_ESM.docx]

**Supplementary Material to:**

**A comparison of eleven clinical risk scores for prediction of survival, after curative-intent resection of colorectal liver metastases.**

Felix Schmidt^1^, Franziska A. Meister^1^, Lea Hitpass^2^, Theresa H. Wirtz^3^, Sven A. Lang^4^, Felix Oldhafer^1^, Oliver Beetz^1^, Martin W. von Websky^1^, Thomas Vogel^1^, Florian W. R. Vondran^1^, Katharina Joechle^#1^ and Iakovos Amygdalos^#1^

**Affiliation:**

^1^University Hospital RWTH Aachen, Department of General, Visceral, Pediatric, and Transplantation Surgery,

^2^University Hospital RWTH Aachen, Department of Diagnostic and Interventional Radiology,

^3^University Hospital RWTH Aachen, Department of Internal Medicine III,

^4^Johannes Wesling University Hospital Minden, Ruhr University Bochum, Department of General, Visceral, Thoracic, and Endocrine Surgery,

**Correspondence to:** Priv.-Doz. Iakovos Amygdalos MBBS PhD, University Hospital RWTH Aachen, Department of General, Visceral, Pediatric, and Transplantation Surgery, Pauwelsstraße 30, 52074 Aachen, Germany, [iamygdalos@ukaachen.de](mailto:iamygdalos@ukaachen.de)

**Running title:** Survival CRS for patients with CRLM

**Supplementary Table 1.** Detailed Points For The Basingstoke Predictive Index

Factors are weighted by the β-coefficient of regression analyses and multiplied by 10

| Risk Factors | Preoperative Score | Postoperative Score |
| --- | --- | --- |
| Primary tumor LN status  Negative  Positive | 0  2 | 0  2 |
| Primary tumor differentiation  Well  Moderate  Poor | 0  3  5 | 0  2  4 |
| CEA level at hepatectomy  < 6 ng/mL  6–60 ng/mL  > 60 ng/mL | 0  2  3 | 0  1  3 |
| No. hepatic metastases  1–3  3 | 0  4 | -  - |
| Largest tumor diameter  < 5 cm  5–10 cm  > 10 cm | 0  2  8 | 0  2  7 |
| Hepatic resection margin  Negative  Positive | -  - | 0  11 |
| Extrahepatic metastatic disease  No  Yes | 0  7 | 0  4 |
